# Supplementary material for: ScaleFusionNet: transformer-guided multi-scale feature fusion for skin lesion segmentation
Source: Sci Rep. 2025 Oct 2;15:34393. doi: 10.1038/s41598-025-17300-x (PMC12491546; doi:10.1038/s41598-025-17300-x)
Supplement: Supplementary file 1 — Supplementary Material [file 41598_2025_17300_MOESM1_ESM.pdf]

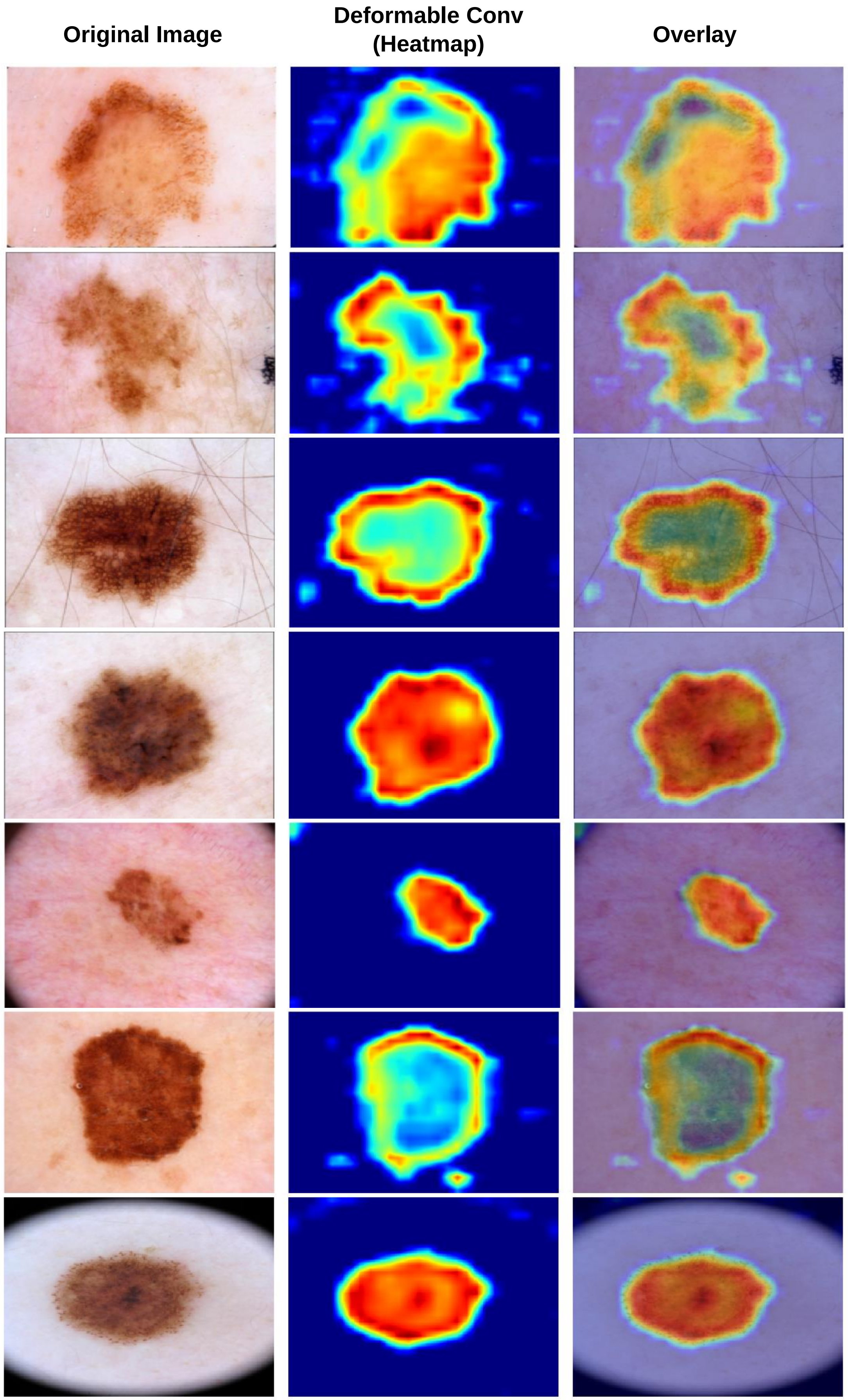

Figure 1. GradCAM heatmap for deformable convolutional layer. Left: original image, middle: heatmap, right: overlay on the image.

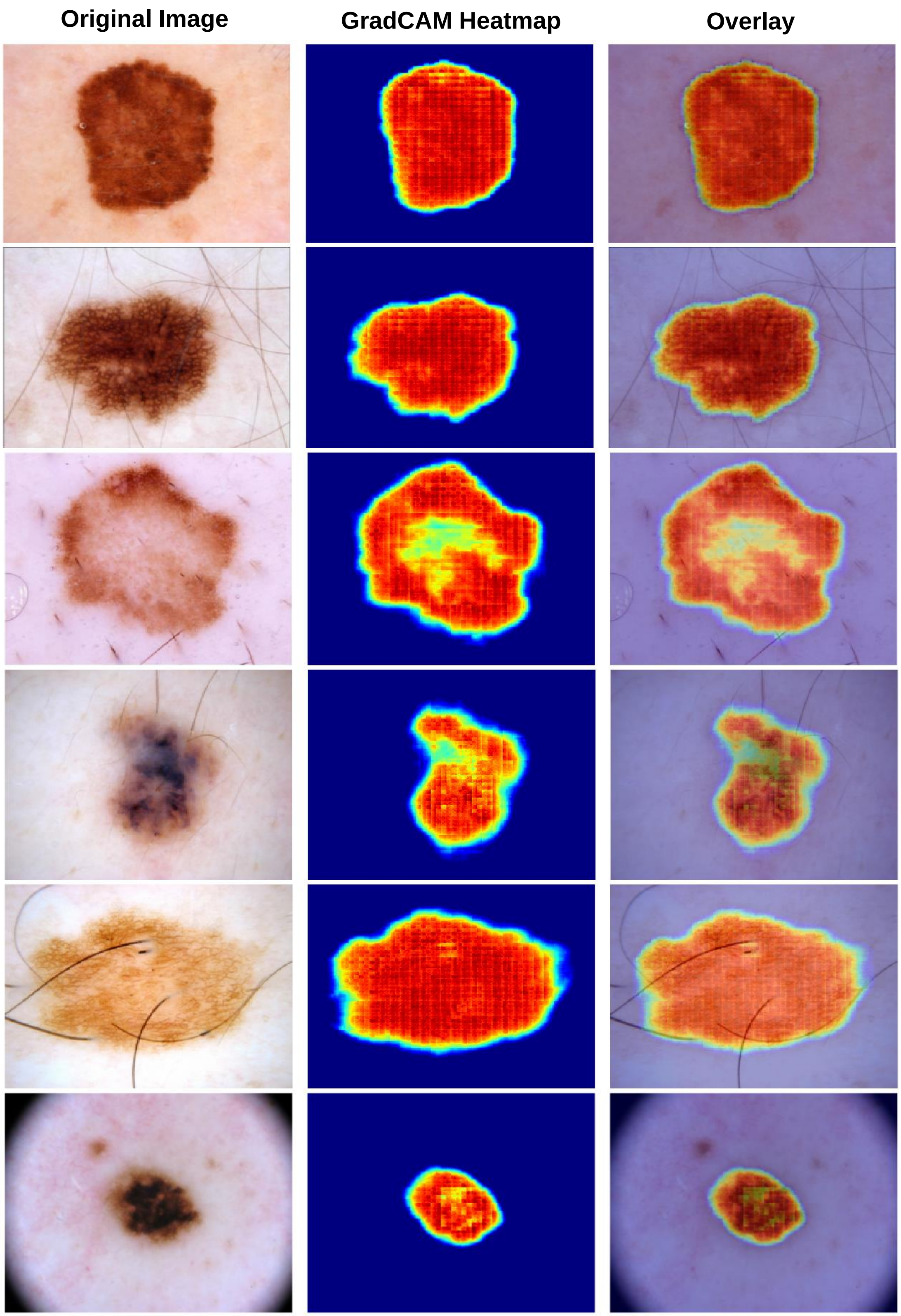

Figure 2. GradCAM heatmap for the last layer showing accurate boundary prediction for skin lesions. Left: original image, middle: heatmap, right: overlay on the image.

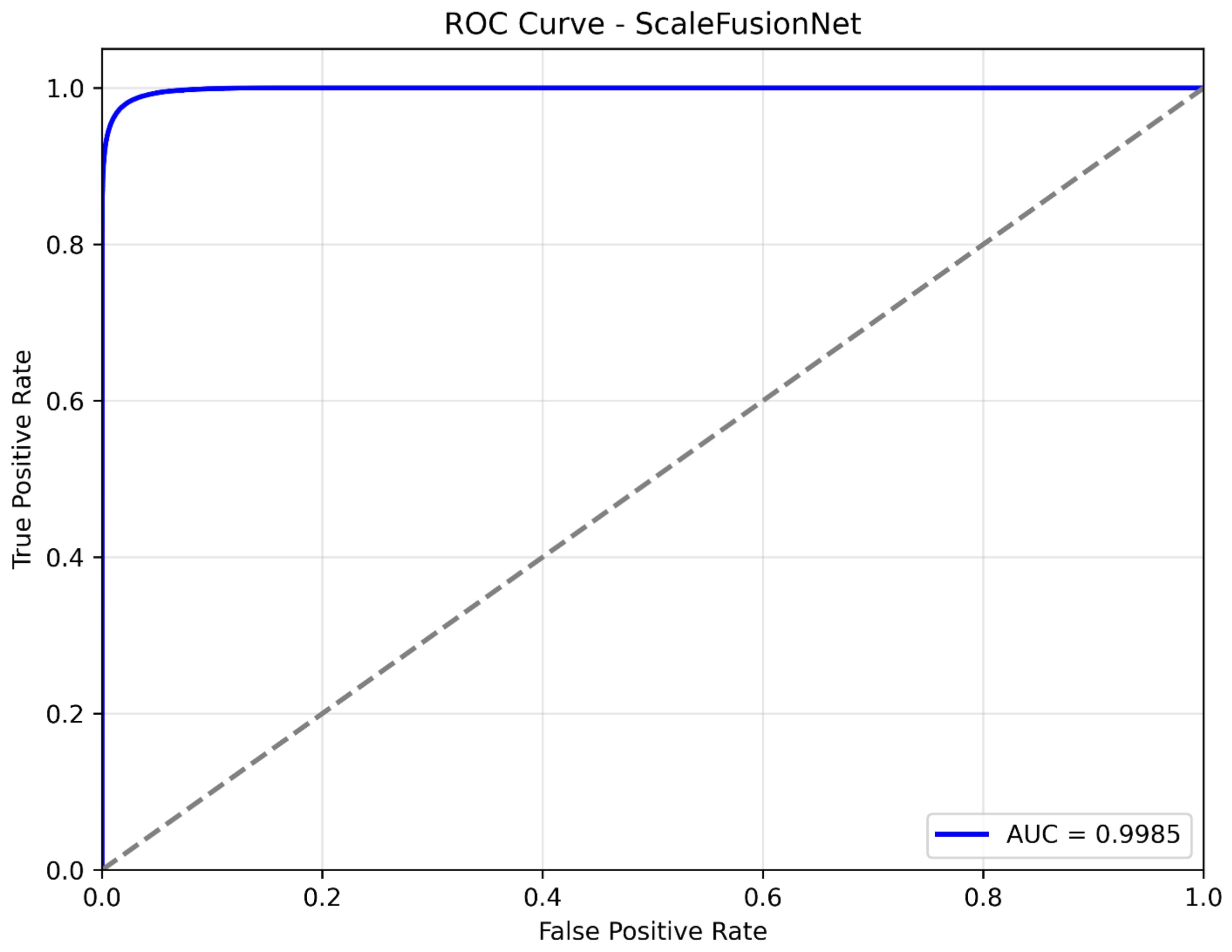

Figure 3. ROC curve of ScaleFusionNet on the ISIC-2018 test set, achieving an AUC of 0.9985, which indicates near-perfect separation between lesion and non-lesion pixels. The curve's proximity to the top-left corner reflects the model's robustness, aligning with its high sensitivity and specificity reported in Table 2.
